# Growth of Infants with Intestinal Failure or Feeding Intolerance Does Not Follow Standard Growth Curves

**DOI:** 10.1155/2017/8052606

**Published:** 2017-03-05

**Authors:** Danielle L. Morton, Keli M. Hawthorne, Carolyn E. Moore

**Affiliations:** ^1^SNG Dialysis, 1520 W. Frank St., Lufkin, TX 75904, USA; ^2^Department of Pediatrics, Dell Medical School at the University of Texas at Austin, 1400 Barbara Jordan Blvd, Austin, TX 78723, USA; ^3^Department of Nutrition and Food Sciences, Texas Woman's University, 6700 Fannin St., Houston, TX 77030, USA

## Abstract

*Objective.* Infants with intestinal failure or feeding intolerance are nutritionally compromised and are at risk for extrauterine growth restriction. The aim of the study was to evaluate growth velocities of infants with intestinal failure and feeding intolerance for the first three months of age and to determine growth percentiles at birth and at 40-week postmenstrual age (PMA).* Methods.* A chart review of infants followed by the Texas Children's Hospital Intestinal Rehabilitation Team was conducted from April 2012 to October 2014. Weekly weight, length, and head circumference growth velocities were calculated. Growth data were compared to Olsen growth curves to determine exact percentiles.* Results.* Data from infants (*n* = 164) revealed that average growth velocities of 3-month-old infants (weight gain, 19.97 g/d; length, 0.81 cm/week; head circumference, 0.52 cm/week) fluctuated and all were below expected norms. At discharge or death, average growth velocities had further decreased (length, 0.69 cm/week; head circumference, 0.45 cm/week) except for weight, which showed a slight increase (weight, 20.56 g/d). Weight, length, and head circumference percentiles significantly decreased from birth to 40-week PMA (*P* < 0.001).* Conclusions.* Growth of infants with intestinal failure or feeding intolerance did not follow standard growth curves.

## 1. Introduction

In the Neonatal Intensive Care Unit (NICU), intrauterine growth curves are the standard method used for assessing weight, length, and head circumference of preterm infants [1]. New intrauterine gender-specific growth curves, known as the Olsen growth curves, were validated and published in 2010. Intrauterine curves are based on cross-sectional birth data from a diverse population and illustrate ideal fetal growth. They differ from longitudinal postnatal curves which reflect actual growth of preterm infants over time.

High-risk infants are commonly classified as small for gestational age (SGA) or large for gestational age (LGA) in the NICU. Infants that are SGA are at risk for adverse outcomes such as inadequate growth and neurodevelopmental delays. Large for gestational age infants are at risk for early hypoglycemia [2] and are more likely to develop metabolic syndrome later in life [3]. Prior to the publication of the Olsen curves, many infants were inaccurately classified as appropriate for gestational age (AGA) when they were in fact SGA or LGA [1]. Therefore, it is possible that some of these infants may not have been evaluated properly for future health risks.

The Olsen growth curves measure gender-specific weight-for-age, length-for-age, and head circumference-for-age of preterm and term infants with gestational ages of 22 to 42 weeks at birth. Gestational age is calculated by the date of the last menstrual period and by examination of the newborn infant using Dubowitz or Ballard scores [4]. When comparing infants to the Olsen growth curves, infants are SGA if they are less than the 10th percentile weight-for-age and LGA if they are greater than the 90th percentile weight-for-age. Infants that are between the 10th percentile and 90th percentile weight-for-age are classified as AGA. Weight is an acute marker of nutritional status, while longitudinal growth reflects chronic nutritional status that may be associated with the overall health status of an infant [5].

Infants with intestinal failure or feeding intolerance are uniquely nutritionally compromised, putting them at high risk for extrauterine growth restriction. Intestinal failure is the result of a critical reduction in the gut's ability to digest or absorb nutrients and occurs when a section of the small intestine does not function properly or is not present secondary to surgery. This can lead to failure to thrive, restricted growth, and/or developmental issues in the neonate. When more than 50% of the small intestine is removed, a significant reduction in both digestion and absorption occurs [6]. The most common form of intestinal failure is short bowel syndrome (SBS), occurring in about 5 infants per million live births [7]. Necrotizing enterocolitis (NEC) is the most common cause of SBS and intestinal failure [7]. This inflammatory condition of unknown etiology is infamous for affecting the preterm and low-birth-weight infant and often requires significant bowel resection [8]. Other common causes of SBS in the United States include resection following intestinal atresia, dysmotility disorder, gastroschisis, or other congenital malformations including midgut volvulus from malrotation [8, 9].

Feeding intolerance is defined as gastric residual volume of more than 50% of the previous feeding volume, emesis, abdominal distension, or both of these symptoms and a decrease, delay, or discontinuation of enteral feedings [10]. Feeding intolerance is the most common gastrointestinal complication seen in preterm infants and often results in withholding enteral nutrition for a period of time which may further stunt growth unless the infant is supported with parenteral nutrition. The exact pathophysiology of feeding intolerance is multifactorial in infants and may be due to immature gastrointestinal motility, delayed gastric emptying, or immature digestion and absorption, all of which are exaggerated in intestinal failure [11].

Although nutritional management strategies to promote appropriate growth in infants with intestinal failure or feeding intolerance have been suggested, little is known about the usual growth patterns for this infant population [12, 13]. Growth of the infant, as reflected by normal weight gain and growth velocity for age when orally and/or enterally fed, is one of the best indicators of full recovery of intestinal function [7]. No data exist, however, documenting the average growth of infants with intestinal failure or feeding intolerance. Furthermore, studies have not assessed the growth trends of these infants in comparison to standardized growth reference curves.

This study was undertaken to determine how well infants with intestinal failure or feeding intolerance grow and thrive over the first three months of life when compared to growth percentiles of an average infant using the Olsen intrauterine gender-specific growth curves.

## 2. Materials and Methods

### 2.1. Participants

Infants were consecutively followed by the Texas Children's Hospital (TCH) Neonatal Intensive Care Unit (NICU) Intestinal Rehabilitation Team from April 2012 to October 2014. Criteria used to assign infants to the Intestinal Rehabilitation Team included short bowel syndrome, feeding intolerance, prolonged parenteral nutrition, or referral by primary physician for reasons such as malabsorption or poor growth. Subjects in this study experienced a wide range of feeding intolerance complications including gastric residual volume of more than 50% of the previous feeding volume, emesis, abdominal distension, or both of these symptoms and a decrease, delay, or discontinuation of enteral feedings. Referral could be for malabsorption, poor growth, or other nutrition related factors such as nutrient deficiencies.

Criteria for inclusion into the study included the following: being followed by TCH rehabilitation team, being admitted to TCH Intestinal Rehabilitation Team before 3 months of age, and survival for at least 7 days of life after being admitted to TCH Intestinal rehabilitation Team. Because the same specialized nutrition support team followed all infants, nutrition practices were similar among infants and feeding volume advancement, conditions to hold feeds, weaning of TPN, use of donor human milk, and use of human milk fortifiers were standardized.

Infants were assigned identification codes to maintain confidentiality and retrospective data were obtained from electronic medical records. Observational data collected from the medical records included medical record number, gender, race, gestational age at birth, date of admission to TCH NICU, date of discharge or death, length of stay, medical and surgical history, primary diagnosis, and weekly anthropometrics.

### 2.2. Anthropometrics and Growth Measures

Weight was recorded daily to the nearest gram, while length and head circumference were recorded to the nearest 0.01 centimeter weekly. Bedside nurses obtained weight using digital infant scales (Scale-Tronix®, Skaneateles Falls, NY). Length and head circumference were measured using a length board (Ellard Instrumentation Ltd., Monroe, WA) and tape measure, respectively. Anthropometric measurements were included if they were obtained and recorded at outside hospitals prior to admission at TCH. A cutoff of 3 months of age was applied when calculating weekly growth velocities during hospitalization. Average weekly growth velocities were compared to growth velocity at time of infant's discharge or death.

Growth velocities were calculated by first recording the weight, length, and head circumference of all the participants every 7 days starting from birth until 3 months of age in order to compare to published standards [1]. Weekly weight, length, and head circumference velocities were then calculated to determine average growth over the past 7 days. Weekly weight gain velocity is calculated by subtracting the previous week's average weight from the current week's average weight and dividing that by seven days to determine average g/d weight gain. Length velocities were also calculated weekly by subtracting the prior week's length from the current week's length to determine cm/wk average length gain. Similarly, weekly head circumference was calculated by subtracting the previous week's head circumference from the current head circumference to determine cm/wk average head circumference gain.

Using the infant's date of birth and gestational age at birth, infants' 40-week PMA date was calculated. This date allowed anthropometric data to be collected at 40-week PMA. Growth velocities were then calculated from birth to 40-week PMA as well as percentiles at 40-week PMA.

The date of discharge or death was recorded and used to calculate the number of days and weeks from birth to discharge or death. These data were used to calculate each infant's growth velocity from birth until discharge or death.

Each infant's growth percentiles for weight, length, and head circumference were determined by comparison to the Olsen curves [1]. When an infant's measurements were beyond the Olsen growth curves (below the 3rd percentile or above the 97th percentile), a one-sided *Z*-score was computed using the number of standard deviations the infant was above or below the mean percentile for gestational age. This *Z*-score was then converted to a percentile.

### 2.3. Statistical Methods

Expected weight gain velocity was approximately 20–30 g/d [14]. Expected length and head circumference growth was approximately 1 cm/wk [14]. Paired *t*-tests were used to compare birth and 40-week PMA percentiles of infants. Statistical significance was defined as *P* < 0.05. Analyses were completed using IBM SPSS Statistics for Windows, Version 19.0 (SPSS Inc., Armonk, NY). All data are mean ± standard deviation unless otherwise noted.

## 3. Results

### 3.1. Characteristics of the Sample

**Figure 1:**
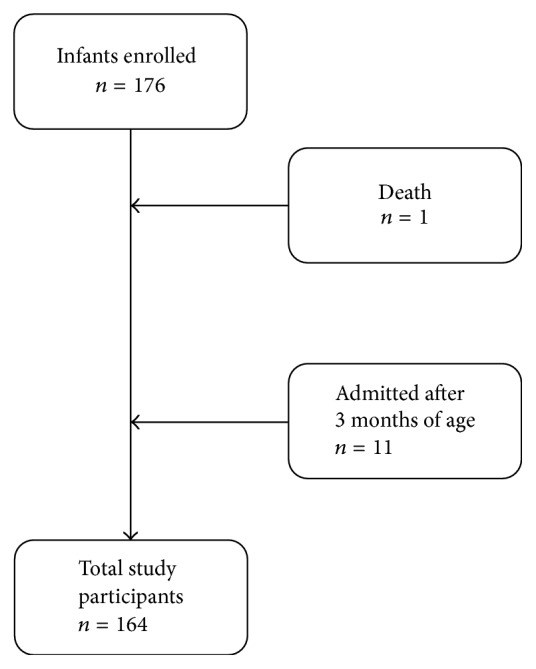
Subject flow diagram.

A total of 176 infants followed by the NICU Intestinal Rehabilitation Team at TCH were enrolled for this study. Of the 176 infants, one infant died before day of life 7 and 11 infants were admitted after 3 months of age, resulting in 164 infants (Figure 1).

Gender distribution of participants was primarily males (62%) versus females (38%) (Table 1). The majority of infants were White or Hispanic infants, followed by Black and Asian infants (Table 1). The average gestational age at birth for this population was 31.5 ± 5.4 weeks and ranged from 23 to 40 weeks (Table 1). The mean birth weight was 1723 ± 951 g, mean birth length was 39.7 ± 7.3 cm, and mean birth head circumference was 27.7 ± 4.6 cm. Mean day of life when admitted to the TCH NICU was 17.1 ± 33.2 days and the average length of stay was 112.6 ± 79.7 days.

### 3.2. Growth Velocities

Growth velocities of this infant population do not represent constant growth over time. Average growth velocity at 3 months of age was below the expected norms in weight, length, and head circumference. At 3 months of age, the average weight gain velocity was 19.97 g/d. By discharge or death, the average weight gain velocity (20.6 g/d) was just above the low range of the expected norm. Mean length growth velocity was also below the expected 1 cm/wk. Length growth velocity for the first 3 months of age was 0.81 cm/wk but by discharge or death it had decreased to 0.69 cm/wk. The average head circumference velocity at 3 months of age was 0.52 cm/wk. Head circumference growth velocity also decreased to 0.45 cm/wk by discharge or death.

### 3.3. Weight, Length, and Head Circumference Percentiles

**Figure 2:**
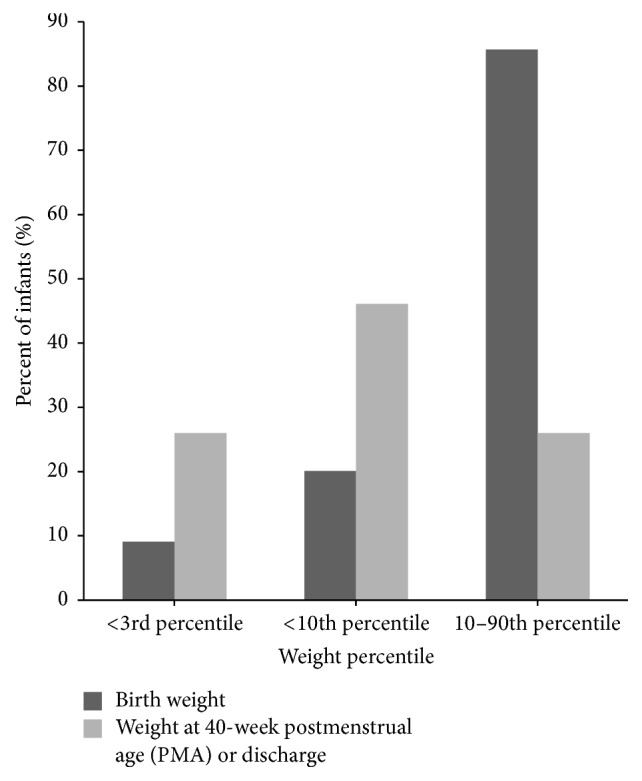
Weight percentile ranking from birth to 40-week postmenstrual age (PMA) or discharge.

Percentile rankings coincided with findings of growth velocities in that they were below average. The mean weight percentile at birth was 43.3 ± 29.2 (Table 2). This percentile significantly decreased to 17.6 ± 21.1  at 40-week PMA (*P* < 0.001). Thirty-one infants (18.9%) were classified as SGA, below the 10th percentile weight-for-age, at birth, while 71 (46.1%) were classified as SGA at 40-week PMA. Similarly, the number of infants below the 3rd percentile increased from 14 (8.5%) to 40 (26%) at 40-week PMA (Figure 2). The number of infants less than the 10th percentile (*n* = 31) and less than the 3rd percentile (*n* = 14) more than doubled (*n* = 71 and *n* = 40, resp.) from birth to 40-week PMA. Infants between the 10th percentile and 90th percentile decreased from 132 (80.5%) to 40 (26%) at 40-week PMA (Figure 2).

**Figure 3:**
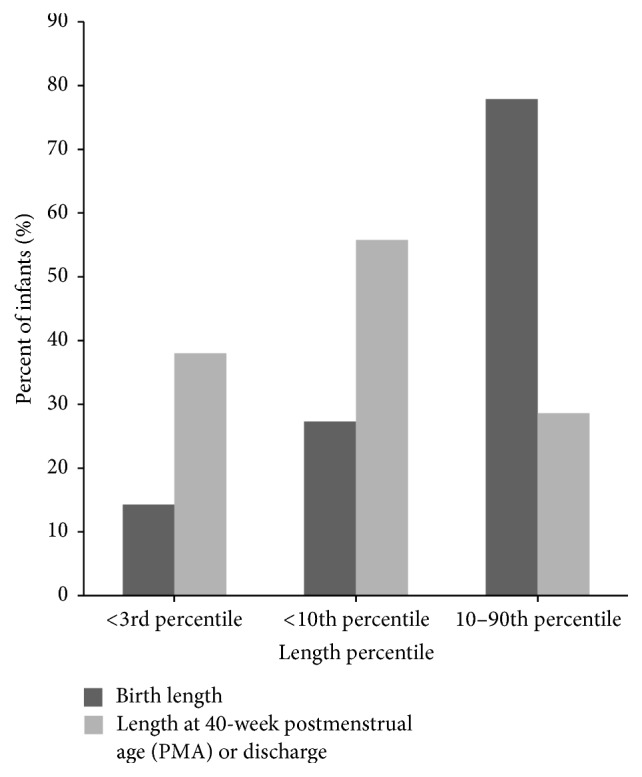
Length percentile ranking from birth to 40-week PMA or discharge.

Length-for-age percentile reduction during hospitalization was similar to the change in weight percentile. Mean length-for-age percentile at 40-week PMA was significantly lower than the mean length-for-age percentile at birth (Table 2) (12.8 ± 19 percentile versus 38.1 ± 29.9 percentile, resp.; *P* < 0.001). Forty-two infants (25.6%) were below the 10th percentile at birth and therefore were considered at nutritional risk. Of these 42 infants, 40 (95.2%) remained below the 10th percentile at 40-week PMA. Twenty-two (13.4%) infants were below the 3rd percentile length-for-age at birth, which increased to 59 infants (38.3%) below the 3rd percentile at 40-week PMA (Figure 3). At birth, 120 infants (73.2%) were between the 10th percentile and 90th percentile. The number of infants between the 10th percentile and 90th percentile significantly decreased to 44 (28.6%) at 40-week PMA.

**Figure 4:**
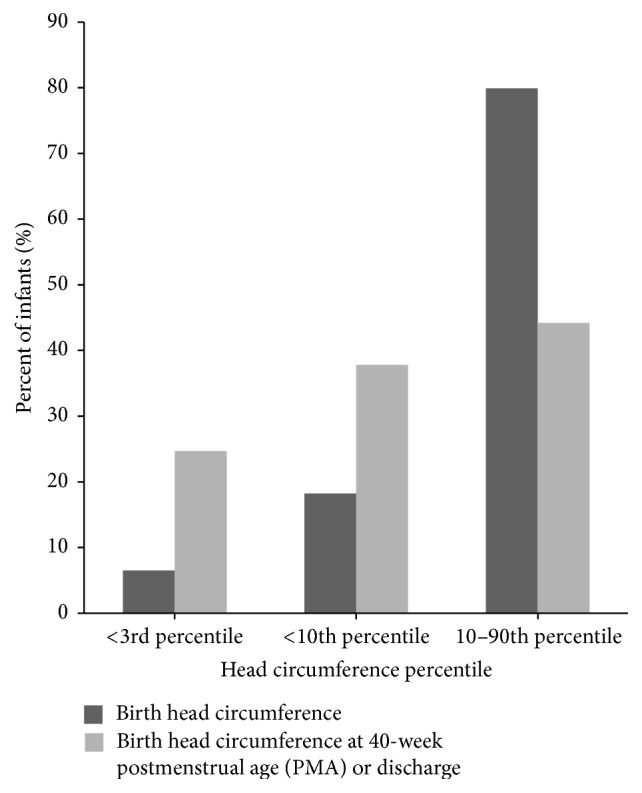
Head circumference percentile ranking from birth to 40-week postmenstrual age (PMA) or discharge.

The difference between the mean head circumference-for-age percentiles at birth and 40-week PMA or discharge was also significant (*P* < 0.001), although the percentile reduction was smaller as compared to weight and length (Table 2). At birth, the mean head circumference-for-age percentile was 41.1 ± 28.7, decreasing to 27.1 ± 28.4 at 40-week PMA or discharge. Ten infants (6.1%) were below the 3rd percentile for head circumference-for-age at birth (Figure 4). At 40-week PMA, 38 infants (24.7%) were below the 3rd percentile for head circumference-for-age. The number of infants in the 10th percentile to 90th percentile range for head circumference-for-age decreased from 123 infants (75%) at birth to 68 infants (44.2%) at 40-week PMA or discharge.

## 4. Discussion

To our knowledge, this is the first study to evaluate the growth of infants with intestinal failure or feeding intolerance. All infants showed a significant decrease of weight, length, and head circumference percentiles from birth to 40-week PMA. By 40-week PMA, more infants were below the 3rd percentile than infants at birth.

### 4.1. Growth Velocity Variation

Although growth velocities of normal infants typically are relatively constant over time, the growth velocities of the NICU infants varied widely [15]. The mean weight growth velocity of 19.97 g/d from birth to 3 months of age had a weekly average range of 1.54 to 28.49 g/d. The mean head circumference velocity of 0.52 cm/wk at 3 months of age was about half the expected normal velocity and ranged from −0.07 to 1.14 cm/wk. The mean length velocity at 3 months of age was 0.81 cm/week with an average weekly range of 0.07 to 1.32 cm/wk. Etiologies of slow growth may be multifactorial and involve not only nutritional management which was closely regulated in this study but also medications, medical procedures, and complications, among others. Consequences of long-term undernutrition resulting from intestinal failure and feeding intolerance in premature infants are not well described but may likely lead to slower cognitive, motor, and communication delays.

Weight, length, and head circumference velocities of infants with intestinal failure and feeding intolerance were below the average of reference infants without intestinal complications at 3 months of age. By discharge, growth velocities had fallen even further from the expected norms in all categories except weight. Weight velocity slightly increased during hospitalization from 3 months of age to discharge.

### 4.2. Reduced Growth

More infants were below the 10th percentile for weight at 40-week PMA compared to birth, placing them at greater health and nutritional risk. The most stunted growth parameter was head circumference, which was almost half the expected rate. Length velocity was also significantly below the expected growth rate for healthy infants. Mean weight gain was the least affected growth parameter. According to Rogol et al., weight is an acute marker of nutritional status, while length may be associated with the long-term health condition of the infant and reflects chronic nutritional status [5]. Thus, the longitudinal measurements in this study reflected a poor overall health condition in this infant population.

The World Health Organization (WHO) growth standard charts are used to calculate growth percentiles of term infants with gestational age of 40 weeks or older. Of the 164 participants in this study, only 6 met the birth gestational age criteria to be compared to the WHO charts; therefore, only the Olsen growth curves were used to obtain percentiles.

The major strength of the study was the large and diverse infant sample size. At least 5 different races/ethnicity groups were represented. In addition, trained nurses were used to measure the infants. Furthermore, by including only infants being followed by TCH NICU Intestinal Rehabilitation Team, standardized nutrition and medical care were provided for infants with intestinal failure and feeding intolerance. Nevertheless, by studying the growth of infants at one hospital in Texas, findings may not be generalized to other NICU infant populations. Clinicians at other facilities may follow different procedures resulting in different outcomes. Thus, more studies are needed to determine causes of slower growth rates and failure to thrive of NICU infants with intestinal failure or feeding intolerance. Furthermore, although some mode of feeding data (not shown) was collected, additional studies are warranted to determine if parenteral or enteral feeding affects growth differently.

## 5. Conclusions

We found that growth was limited among infants with intestinal failure and feeding intolerance even when being followed by a specialized nutrition support team. Future studies should identify specific interventions to improve the growth of infants with intestinal failure or feeding intolerance. Expanding the study to other research sites would increase the knowledge base and may help identify successful approaches to help these infants thrive and grow.

## Figures and Tables

**Table 1:** Demographic characteristics of NICU infants with intestinal failure or feeding intolerance (*N* = 164).

Characteristic	Frequency (*n*)	Percentage (%)	Mean ± SD
*Gender of child*			
Male	102	62	
Female	62	38	
*Ethnicity*			
White	65	39.6	
Hispanic	47	28.7	
Black	37	22.6	
Asian	12	7.3	
Other	3	1.8	
*Age of infants*			
Birth gestational Age (wks)			31.5 ± 5.4
*Anthropometrics*			
Birth weight (g)			1723 ± 951
Birth length (cm)			39.7 ± 7.3
Birth head circumference (cm)			27.7 ± 4.6
*Hospitalization*			
Admission day of life (d)			17.1 ± 33.2
Average length of stay (d)			112.6 ± 79.7

**Table 2:** Change in anthropometric percentiles from birth to 40-week PMA among NICU infants with intestinal failure or feeding intolerance (*N* = 164).

Variable	Birth Mean ± SD	40-week PMA Mean ± SD	Change	*P* value^*∗*^
Weight-for-age percentile	43.3 ± 29.2	17.6 ± 21.1	−25.7 ± 25.6	*P* < 0.001
Length-for-age percentile	38.1 ± 29.9	12.8 ± 19.0	−25.3 ± 27.2	*P* < 0.001
Head circumference-for-age percentiles	41.1 ± 28.7	27.1 ± 28.4	−14.0 ± 2.7	*P* < 0.001

^*∗*^Significant at *P* < 0.05.

## Abbreviations

AGA: Appropriate for gestational age
LGA: Large for gestational age
NEC: Necrotizing enterocolitis
NICU: Neonatal Intensive Care Unit
PMA: Postmenstrual age
SBS: Short bowel syndrome
SGA: Small for gestational age
TCH: Texas Children's Hospital
WHO: World Health Organization.
